# Cecal Ligation and Puncture Results in Long-Term Central Nervous System Myeloid Inflammation

**DOI:** 10.1371/journal.pone.0149136

**Published:** 2016-02-10

**Authors:** Benjamin H. Singer, Michael W. Newstead, Xianying Zeng, Christopher L. Cooke, Robert C. Thompson, Kanakadurga Singer, Ramya Ghantasala, Jack M. Parent, Geoffrey G. Murphy, Theodore J. Iwashyna, Theodore J. Standiford

**Affiliations:** 1 Department of Internal Medicine, Division of Pulmonary and Critical Care Medicine, University of Michigan Medical School, Ann Arbor, Michigan, United States of America; 2 Molecular and Behavioral Neuroscience Institute, University of Michigan, Ann Arbor, Michigan, United States of America; 3 Department of Pediatrics, Division of Endocrinology and Metabolism, University of Michigan Medical School, Ann Arbor, Michigan, United States of America; 4 Department of Neurology, University of Michigan Medical School, Ann Arbor, Michigan, United States of America; 5 Department of Molecular and Integrative Physiology, University of Michigan Medical School, Ann Arbor, Michigan, United States of America; 6 Center for Clinical Management Research, VA Ann Arbor Health System, Ann Arbor, Michigan, United States of America; Indiana School of Medicine, UNITED STATES

## Abstract

Survivors of sepsis often experience long-term cognitive and functional decline. Previous studies utilizing lipopolysaccharide injection and cecal ligation and puncture in rodent models of sepsis have demonstrated changes in depressive-like behavior and learning and memory after sepsis, as well as evidence of myeloid inflammation and cytokine expression in the brain, but the long-term course of neuroinflammation after sepsis remains unclear. Here, we utilize cecal ligation and puncture with greater than 80% survival as a model of sepsis. We found that sepsis survivor mice demonstrate deficits in extinction of conditioned fear, but no acquisition of fear conditioning, nearly two months after sepsis. These cognitive changes occur in the absence of neuronal loss or changes in synaptic density in the hippocampus. Sepsis also resulted in infiltration of monocytes and neutrophils into the CNS at least two weeks after sepsis in a CCR2 independent manner. Cellular inflammation is accompanied by long-term expression of pro-inflammatory cytokine and chemokine genes, including TNFα and CCR2 ligands, in whole brain homogenates. Gene expression analysis of microglia revealed that while microglia do express anti-microbial genes and damage-associated molecular pattern molecules of the *S100A* family of genes at least 2 weeks after sepsis, they do not express the cytokines observed in whole brain homogenates. Our results indicate that in a naturalistic model of infection, sepsis results in long-term neuroinflammation, and that this sustained inflammation is likely due to interactions among multiple cell types, including resident microglia and peripherally derived myeloid cells.

## Introduction

Sepsis, a syndrome of infection and systemic inflammatory response, is a leading cause of mortality in hospitalized patients [1]. As care for critically ill patients has improved, sepsis survival rates have increased [2]. In the United States alone, these trends have resulted in more than 200,000 new long-term sepsis survivors every year [3]. As this cohort of sepsis survivors has grown, so has the recognition that the burdens of survivorship include long-term cognitive impairment, mood disorders, and post-traumatic stress [4–8]. While the strains of critical illness, including hypoxia, hypoperfusion, and metabolic dysfunction likely play a role in cognitive impairment and neuropsychiatric disorders after sepsis, concerns about long-term quality of life are often superseded by life-saving care during the acute illness period. Therefore, pathologic processes that continue during the period of convalescence from sepsis are ideal targets for reducing the burdens of sepsis survivorship.

The central nervous system response to sepsis has been extensively investigated using systemic administration of high-dose lipopolysaccharide (LPS), as a model of sepsis. This model produces both deficits in learning and memory and increased depressive behavior for at least 30 days following the systemic inflammatory insult [9,10]. Acutely, LPS injection results in CNS infiltration of monocytes and neutrophils, as well as increased expression of pro-inflammatory cytokines and chemokines [11–13]. LPS produces dramatic activation of microglia, the innate myeloid lineage cell of the central nervous system [14–16]. A single administration of LPS has also been shown to set long-term neuroinflammatory and neurodegenerative processes in motion, with chronic microglial activation, cortical and subcortical neuronal loss, and changes in synaptic dynamics [10,14,17–19]. A significant question underlying these models, however, is to what degree a single high-dose administration of LPS mimics the inflammatory stimuli presented to the immune system during sepsis. Although LPS signaling is mediated by a single receptor, the intracellular signaling networks engaged by that stimulus depend on dose, timing, and duration of exposure [20]. Indeed, varying doses of LPS have been shown to have neuroprotective effects [21,22]. How the neuroinflammatory response to single-dose LPS relates to the complex balance of tolerance, priming, and activation likely involved in the innate immune response to infection, then, is unclear.

Cecal ligation and puncture (CLP) is a reproducible, titratable, naturalistic model of abdominal sepsis in which intra-abdominal infection from controlled bowel perforation leads to systemic inflammation [23]. Severe CLP, which results in less than 50% survival, leads to multiple neurocognitive deficits in processes such as novel object recognition, spatial learning, and inhibitory avoidance which persist up to 30 days post CLP but resolve in the two months following sepsis [24–27]. Milder CLP, with greater than 70% survival, has been shown to lead to deficits in spatial learning [28]. Acutely, CLP results in morphologic activation of microglia and astrocytes, as well as increased levels of TNFα and IL-6 [29,30]. Septic rodents also undergo acute changes in leukocyte adhesion and microvascular permeability in the brain [31–33]. However, the cellular and molecular substrates of long-term neuroinflammation after CLP are less clear. In particular, whether infiltrating leukocytes, microglia, astrocytes, or the microvasculature are the source of inflammatory mediators has important implications for the design of potential neuromodulatory or immunomodulatory therapies for brain dysfunction after sepsis.

Here, we hypothesize that sepsis results in persistent neuroinflammation over weeks to months. Using a form of CLP with high (>80%) survival, we have found that CLP results in persistent deficits in extinction learning 50 days after CLP. These changes occurred in the absence of overt increases in cell death or synaptic structure in the hippocampus. CLP did, however, result in long lasting vasculopathy, elevation in brain cytokines, and CNS myeloid inflammation. While microglia do not appear to be the source of these cytokines, transcriptome analysis does indicate that CLP results in distinct changes to microglial gene expression. These results highlight the importance of chronic neuroinflammation after sepsis even in the absence of ongoing overt neuronal death. The neuroinflammatory response after polymicrobial sepsis likely depends on interactions between multiple immunologically active cell types, and is not a simple function of microglial activation alone.

## Materials and Methods

### Mice

Wild-type mice used in these experiments were male C57BL/6 mice obtained from Jackson Laboratory and used at 8–10 weeks of age. CCR2^-/-^ mice were of the strain B6.129S4-*Ccr2tm1Ifc*/J.

All procedures involving animals were undertaken in strict accordance with the recommendations of the Guide for the Care and Use of Laboratory Animals by the National Institutes of Health. The study protocol was approved by the University Committee on the Use and Care of Animals of the University of Michigan (protocol #PRO00004786).

### Cecal ligation and puncture

Cecal ligation and puncture was performed as previously described [23]. Animal suffering and distressed was minimized by local infiltration of lidocaine to the abdominal wall as well as anesthesia with ketamine and xylazine. A 1–2 cm laparotomy was performed under aseptic conditions. The cecum was ligated with a silk suture and punctured once through-and-through with a 19 gauge needle. The abdominal wall and incision were closed with surgical clips. Imipenem/cilastatin (Merck, 0.5 mg/mouse) and normal saline (1 mL) were administered subcutaneously at the time of surgery. Sham operated animals underwent laparotomy without ligation or puncture of the cecum, and received antibiotics and saline. Mice were monitored continuously until recovery from anesthesia, and at least twice per day during the 10 days after surgery. The University of Michigan policy for humane endpoints was followed, and animals were euthanized by husbandry or laboratory staff if found to be moribund, immobile, unable to access food or water, or with wound dehiscence or bleeding. Euthanasia was performed with CO_2_ inhalation in this circumstance. All deaths occurred within 5 days of CLP, were attributed to the procedure, and were considered expected.

### Pavlovian fear conditioning

Mice were tested in one of four chambers equipped with a stainless steel floor grid designed for mice (Med Associates). The chambers were arranged in a 2 x 2 grid in an isolated room separate from the room where mice were housed. Each chamber was equipped with an overhead camera, with video signals sent to Actimetrics FreezeFrame software. Foot shock generation and auditory tones were controlled by the same software. Freezing was quantified by the Actimetrics FreezeFrame software based on frame-by-frame analysis of the video signals, digitized at 4 Hz. Testing was performed at the same time each day.

#### Contextual fear conditioning

The conditioned context was defined by placing 95% ethanol in a pan below each grid, lighting the room with white light, and auditory stimulation with white noise. Mice were placed individually in each chamber, and four mice were tested at a time. During conditioning, mice were placed in the chamber 3 minutes prior to the onset of a 30 second tone. At the end of the tone, mice received the unconditioned stimulus (2 second 0.5 mA foot shock). Mice remained in the chamber for an additional 30 seconds and were then removed and returned to their home cages. Conditioning was performed once daily for a total of 3 sessions on consecutive days. On the fourth day, generalization of the freezing response and the conditioned response to tone were tested by placing mice in novel context, defined by placing 1% acetic acid in the pan below each grid, lighting the room with red light, and removing the white noise. Response to the novel context was measured by freezing during 3 minutes prior to the tone being played. The tone was played 3 times at 30 second intervals, and freezing in response to tone was averaged over the interval after each tone. On the fifth day, contextual fear conditioning was measured by freezing when mice were placed back into the conditioned context for 3 minutes.

#### Trace tone conditioning

Experiments were carried out in the same manner as contextual fear conditioning, with the exception that a 30 second trace interval was introduced between the tone and unconditioned stimulus.

#### Extinction of conditioned fear

Extinction was measured starting on the eighth day after the initiation of fear conditioning. Mice were placed back in the conditioned context for 30 minutes without any additional stimuli. Freezing was quantified over the entire session. Extinction continued for four consecutive days.

### RNA isolation from brain homogenates

Mice were deeply anesthetized by CO_2_ inhalation, and trascardially perfused with ice-cold phosphate buffered saline (PBS). The brain was then removed and immediately frozen in liquid nitrogen, and stored at -80°C. Whole brain mRNA was prepared by homogenization in Trizol (Invitrogen), followed by centrifugation to remove fatty debris, extraction in chloroform, and isopropanol precipitation as per the manufacturer’s instructions.

### RT-PCR

Real-time RT-PCR was performed on an ABI Prism 7000 thermocycler (Applied Biosystems, Foster City, CA) and analyzed as previously described [34]. Gene-specific primers and probes were purchased from Integrated DNA Technologies (Coralville, IA).

### Flow cytometry

Single cell suspensions were prepared as previously described [35]. Briefly, mice were deeply anesthetized with CO2 and transcardially perfused with ice-cold HBSS. Brains were grossly free of blood after perfusion. Brains were minced, mechanically triturated and passed through a 70 μm cell strainer to form a single cell suspension. Leukocytes were enriched by centrifugation over a discontinuous gradient of 30%, 37%, and 70% Percoll and collecting the fraction at the 37%/70% boundary. Cells were then washed and stained with fluorophore conjugated antibodies as indicated in the text and figures, followed by fixation in paraformaldehyde prior to analysis on an FACSAria II flow cytometer. Antibodies include anti-CCR2 (clone 475301, R&D Systems), anti-CD11b (clone M1/70, BD), anti-CD45 (clone 30-F11, BD), anti-CD64 (clone X54-5/7.1), anti-CX3CR1 (goat anti-mouse polyclonal, R&D Systems), anti-Ly6G (clone 1A8, Biolegend), and anti-Ly6C (clone HK1.4, Biolegend).

### Cell sorting and gene expression analysis

Single cell suspensions were prepared by homogenization with enzymatic digestion (Miltenyi), stained with fluorophore conjugated antibodies, treated with propidium iodide as a live/dead marker, and sorted by FACS. Brains from at least 3 mice were pooled per experimental condition. Microglia were identified as CD45^mid^/CD11b^+^/CD64^+^ cells, and monocytes as CD45^hi^/CD11b^+^/Ly6C^+^/Ly6G^-^ cells. Cells were sorted directly into Trizol and stored at -80C until extraction by chloroform and isopropanol precipitation. RNA concentration was quantified by spectrophotometry (NanoDrop 2000, Thermo) and quality was assessed by chip electrophoresis (BioAnalyzer PicoChip, Agilent). Preparation of cDNA, probe labeling, and hybridization to Affymetrix Mouse Genome 430 2.0 arrays was performed by the University of Michigan Microarray Core Facility. Expression values were derived using robust multiarray averages in the Bioconductor suite implemented in the R statistical environment [36].

### Histology

Mice were euthanized and transcardially perfused with PBS as above. Mice were subsequently perfused with 4% buffered paraformaldehyde. Brains were then removed and post-fixed in PFA for 4 hours at 4°C, briefly rinsed and immersed in buffered 30% sucrose solution until they were no long buoyant. Cryoprotected tissues were frozen on dry ice and stored at -80°C until cut into 40 μm sections using a cryostat (Leica).

#### Nissl staining

Sections were mounted on Superfrost Plus slides (Fisher). Fat was cleared in graduated ethanol solution followed by xylene, and tissue sections were rehydrated in graduated ethanol solutions and water. Slides were then stained in 1% cresyl violet and destained in acetic acid followed by dehydration, and then coverslipped in Permount (Sigma). The volume of the ventral hippocampal stratum pyramidale was estimated by measuring the area of the layer in the three most ventral hippocampal sections in a series of every 12^th^ section. This area was summed and multiplied by the nominal inter-section distance of 480 μm to obtain an estimated volume.

#### FluoroJade

Sections were mounted and stained on gelatin-subbed slides with FluoroJade C (Histo-Chem) according to the manufacturer’s instructions. Briefly, sections were treated with 1% NaOH in 80% EtOH, followed by rinses in 70% EtOH and water. Sections were then treated with 0.06% KMnO_4_ solution and stained in 0.0001% FluoroJade in 1% acetic acid, rinsed, dehydrated in xylene, and coverslipped in DPX mounting medium.

#### Immunohistochemistry

Sections were processed using a free-floating technique. Endogenous peroxidases were deactivated by treatment with 1% H_2_O_2_, followed by permeabilization with 0.1% Triton-X and 0.05% BSA in Tris buffer. Sections were then blocked in 10% normal goat serum and washed. Sections were incubated with anti-CCR2 antibody (R&D Systems, clone 475301, rat monoclonal antibody raised to full length mouse CCR2 expressed in a L1.2 pro B-cell line, detects HEK cells transfected with CCR2 but not CCR5 by flow cytometry) overnight at 4°C. Sections were then washed and incubated with biotinylated horse anti-rat IgG secondary antibody (Vector) for 90 minutes at room temperature. The secondary antibody signal was amplified by incubation with avidin-biotin complex (Vector) and developed using diaminobenzidine (Invitrogen). Sections were then mounted and coverslipped in Permount.

#### Immunofluorescence

For detection of Iba1, sections were blocked and permeabilized by incubation with blocking buffer (10% normal goat serum, 3% BSA, 1% glycine, 0.4% Triton-X in Tris-buffered saline). Sections were incubated in rabbit anti-Iba1 (Dako, rabbit polyclonal antibody raised to a synthetic peptide corresponding to the C-terminus of Iba1, identifying a single 17 kDa band on Western blot of microglial lysates) overnight at 4°C. For staining of C1q, sections were incubated with rabbit anti-C1q (Epitomics, rabbit monoclonal antibody raised against full length mouse C1q with specificity determined by immunofluorescence in C1q knockout mice [37]). Sections were then washed and incubated at room temperature with Alexa-488 conjugated anti-rabbit secondary antibody (Invitrogen). Slides were coverslipped in ProLong Gold mounting medium (Invitrogen). For staining of mouse IgG, sections were similarly blocked and permeabilized, and incubated with Alexa-594 conjugated anti-mouse IgG antibody overnight at 4°C. Sections were then rinsed and coverslipped in ProLong Gold.

#### Immunofluorescence intensity measurements

Quantification of immunofluorescence signal was performed on tissue processed and stained in parallel, and imaged in a single session using identical exposure and gain settings. In order to maximize the sensitivity of the measurements, the exposure was checked on all specimens and set to maximize the dynamic range on the brightest specimen. For quantification of C1q staining, multiple images at 20x were taken over the extent of the hippocampus, and mean brightness over the stratum moleculare was calculated in ImageJ. For quantification of Iba1 staining, 3 images over the cortex were taken at 20x in each animal, and the brightness over the entire field was averaged, as previously described [38].

### Golgi staining and dendritic spine density

Golgi impregnation and staining was performed by the Golgi-Cox method using the Rapid GolgiStain kit (FD Neurotechnologies). Mice were euthanized using CO_2_ and brains were dissected without perfusion. Brains were immersed in impregnation solution for two weeks, then rinsed for up to 1 week with the provided rinse buffer. Brains were then frozen and sectioned at 100 μm thickness using a cryostat and mounted directly to gelatin-subbed slides. Sections were then developed and coverslipped in Permount. Dendritic segments were identified in the molecular layer of the dentate gyrus. Dendrites in which at least a 20 μm segment lay in the same plane of focus were photographed using an Olympus BX-51 using a 100x oil immersion objective. The number of dendritic spines and length of dendritic segment were then measured using ImageJ software. At least 5 segments per section, and 5 sections per animal were examined. The investigator was blinded to the treatment group during selection of dendritic segments and spine counting. This method conforms to previously reported techniques for using analysis of 2 dimensional images to estimate dendritic spine density [39].

### Data Analysis

Training in the fear conditioning paradigm was evaluated using a repeated measures ANOVA, and the response to novel context, tone, and context were evaluated with separate one-way ANOVAs testing the effect of treatment group. For extinction of contextual fear conditioning, freezing was evaluated using a general linear model with within-subject factor of day and between subject factor of treatment group. RNA expression data was analyzed by 1-way ANOVA, with each time/treatment combination treated as a separate group. In order to minimize spurious comparisons, we prespecified post-hoc comparisons only among CLP/sham and CLP/unoperated at each time point, and among CLP and CLP at different time points. Flow cytometric data was analyzed by 2-way ANOVA, with factors of time after treatment and treatment group. All post-hoc tests were corrected for multiple comparisons. All figures show mean and standard error unless otherwise specified. Statistical analyses were carried out either in SPSS or GraphPad Prism.

## Results

### CLP results in impaired extinction learning without deficits in fear conditioning

**Fig 1:**
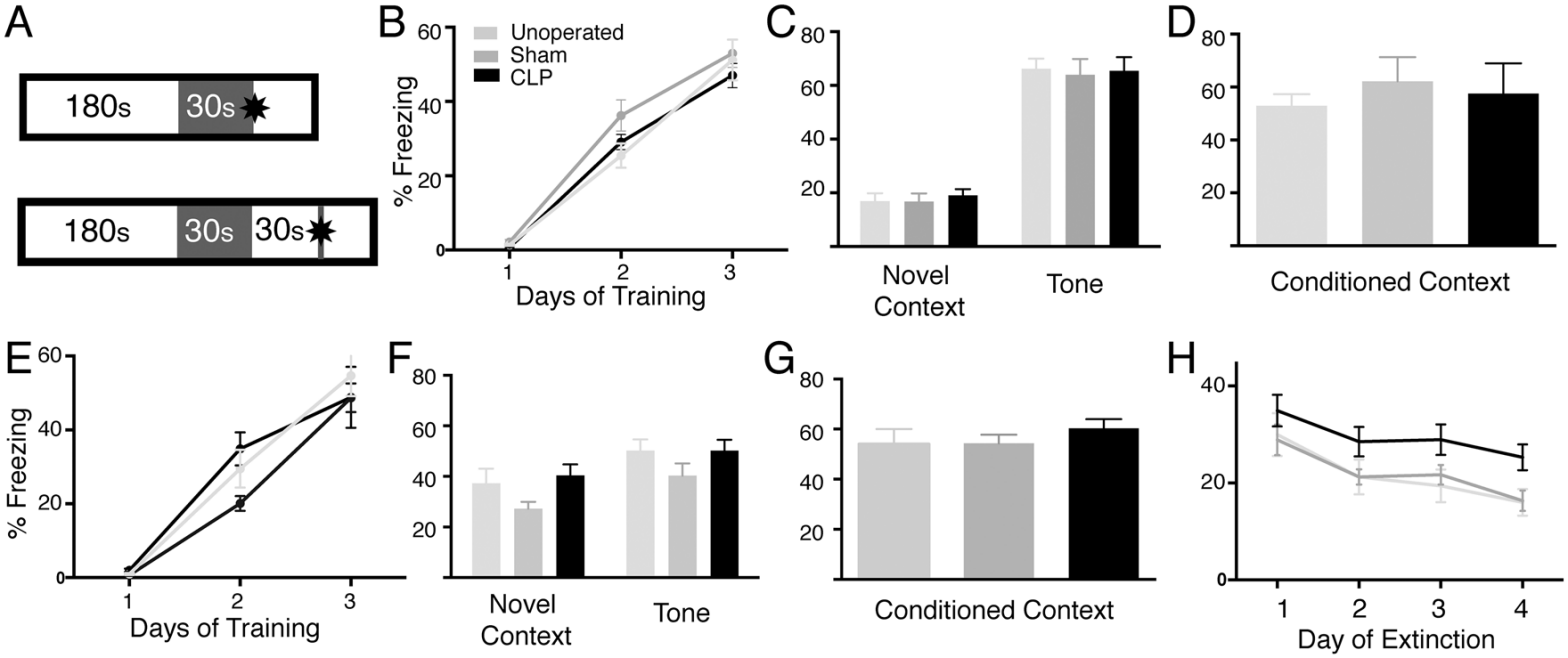
Sepsis survivor mice do not show a deficit in contextual fear conditioning or trace fear conditioning 50 days after CLP, but do demonstrate impaired extinction of conditioned fear. A: In a contextual fear conditioning paradigm, mice were placed in a defined context for 180 s (white bar) at rest. A tone was played for 30 s (gray bar), followed immediately by a foot shock (star). In the trace fear conditioning paradigm, a 30 s delay is introduced after the tone and before the administration of the foot shock. B: In contextual fear conditioning, there is no difference between the rate at which sepsis survivor and control mice associated the freezing response with the context. C: After three days of training, there was no difference in the response of post-CLP and control mice to the tone alone in a novel context. D: There was no difference among post-CLP and control mice in the freezing response to the conditioned context. E-G. In the trace fear conditioning paradigm, there were also no differences among groups in the acquisition of the freezing response, response to tone, or response to context. H. Post-CLP mice demonstrate increased freezing in the conditioned context compared to sham-operated and unoperated mice during repeated exposures without foot shock, indicated a deficit in extinction learning.

We first sought to determine whether CLP results in long-term impairments in learning and memory. We utilize a form a CLP with low mortality (>80% survival) to model the typical survival rate observed in sepsis patients identified from administrative data. Given that the hippocampus is sensitive to metabolic and hemodynamic insults, we examined the effect of CLP on contextual fear conditioning, a form of Pavlovian learning which is dependent upon the hippocampus (Fig 1A) [40]. Mice were tested 50 days after CLP or sham surgery, or were age-matched controls housed under the same conditions. This time point was selected based on previous work which indicated that homeostatic processes in the hippocampus may take up to 2 months to reorganize synaptic structure after a severe acute insult [41]. There was no difference in freezing during the training period (Fig 1B). Mice in all groups froze more in response to tone than to a novel context, indicating acquisition of fear conditioning (Fig 1C and 1F(2,72) = 10.77, *p* < 0.002). There was no difference among groups in freezing in response to tone, which is conditioned in a non-hippocampal dependent manner, or the conditioned context (Fig 1C, 1D and 1F(2,35) = 0.7154, *p* = 0.59).

Given that fear conditioning is a robust form of hippocampal dependent learning, we increased the difficulty of the task by introducing a trace interval between the conditioned and unconditioned stimuli (Fig 1A) [42]. There were no significant among groups in the acquisition of freezing in response to context or tone (Fig 1E), or in freezing in response to tone or context (Fig 1F and 1G). Though there was generalization of freezing in the novel context, this was not specific to the post-CLP group (Fig 1F).

In order to further interrogate whether CLP results in deficits in behaviors which require coordination between multiple brain regions, and are not primarily dependent on the hippocampus, we studied extinction of contextual fear conditioning in the animals which had undergone contextual trace fear conditioning (Fig 1H). All groups showed significant extinction of contextually conditioned fear (F(2,35) = 3.0, *p* < 1 x 10^−5^ for effect of day). However, post CLP animals froze more than sham-operated or untreated mice (*p* = 0.038 for effect of group), indicating a defect in extinction of conditioned fear.

### CLP does not result in ongoing hippocampal cell death or loss of dentate gyrus dendritic spine density

**Fig 2:**
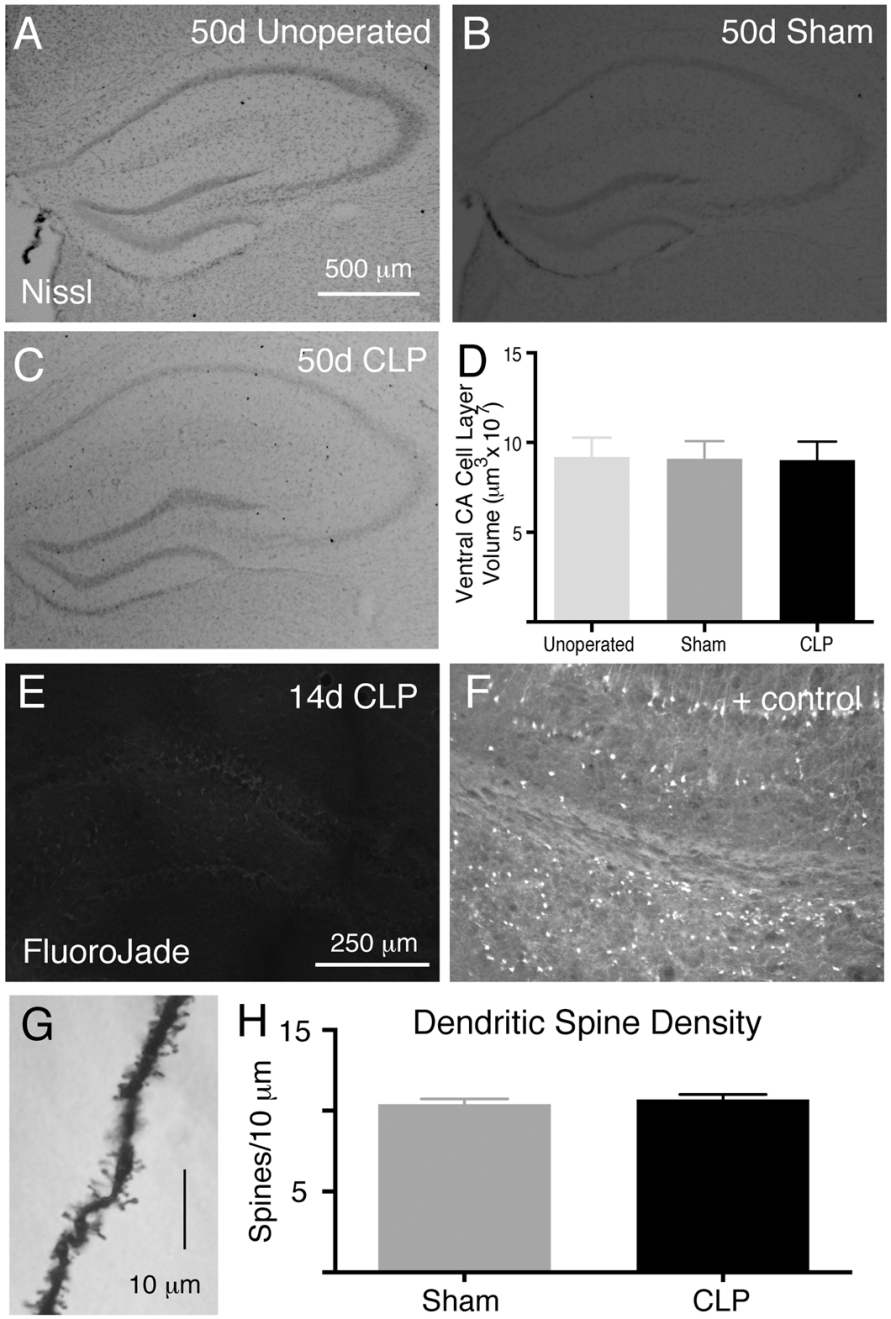
CLP does not result in significant changes to the structure of the hippocampus. There is no difference in the morphology of the hippocampus in 50 days after CLP (C) compared to 50 days after sham operation (B) or in age matched unoperated controls (A). There is no difference in the volume of the ventral stratum pyramidale of the cornu ammonis (D). The lack of change in hippocampal volume 50 days after CLP is concordant with the absence of FluoroJade staining, which necrotic or apoptotic cell death, 14 days after CLP (E). Positive FluoroJade staining in the hippocampus after status epilepticus is shown for comparison (F). Golgi staining was used to determine synaptic density on pyramidal cell dendrites of the dentate gyrus (G). There was no measurable difference among sham operated and post-CLP mice (H).

High dose systemic LPS administration causes death of neurons in the hippocampus [17] We examined hippocampal neuron density by Nissl staining 50 days after low-mortality CLP. There was no gross change in the thickness of the stratum pyramidale layers of the cornu ammonis in post-CLP animals compared to unoperated controls (Fig 2A–2D, n = 4 per group), as had been observed with more severe shock models. Since a low rate of ongoing neuronal death may not be detectable by examining overall cell density, we used FluoroJade staining to detect necrotic an apoptotic cell death [43]. By fourteen days after CLP, there were no detectable FluoroJade positive cells in the hippocampus, as there were in positive control tissue taken from mice which had undergone status epilepticus (Fig 2E and 2F, n = 4), and thus no evidence of ongoing cell death.

Metabolic insults and inflammation may also lead to derangement of synaptic structure without resulting in irreversible cell death, and CLP has been reported to result in long lasting decreases in dentritic spine density [28]. We examined dendritic spine density in the dendrites of pyramidal cells in the molecular layer of the dentate gyrus by the Golgi technique 50 days after CLP (Fig 2G). When examined in blinded fashion, there was no difference in dendritic spine density 50 days after CLP compared to unoperated age-matched controls (Fig 2H).

**Fig 3:**
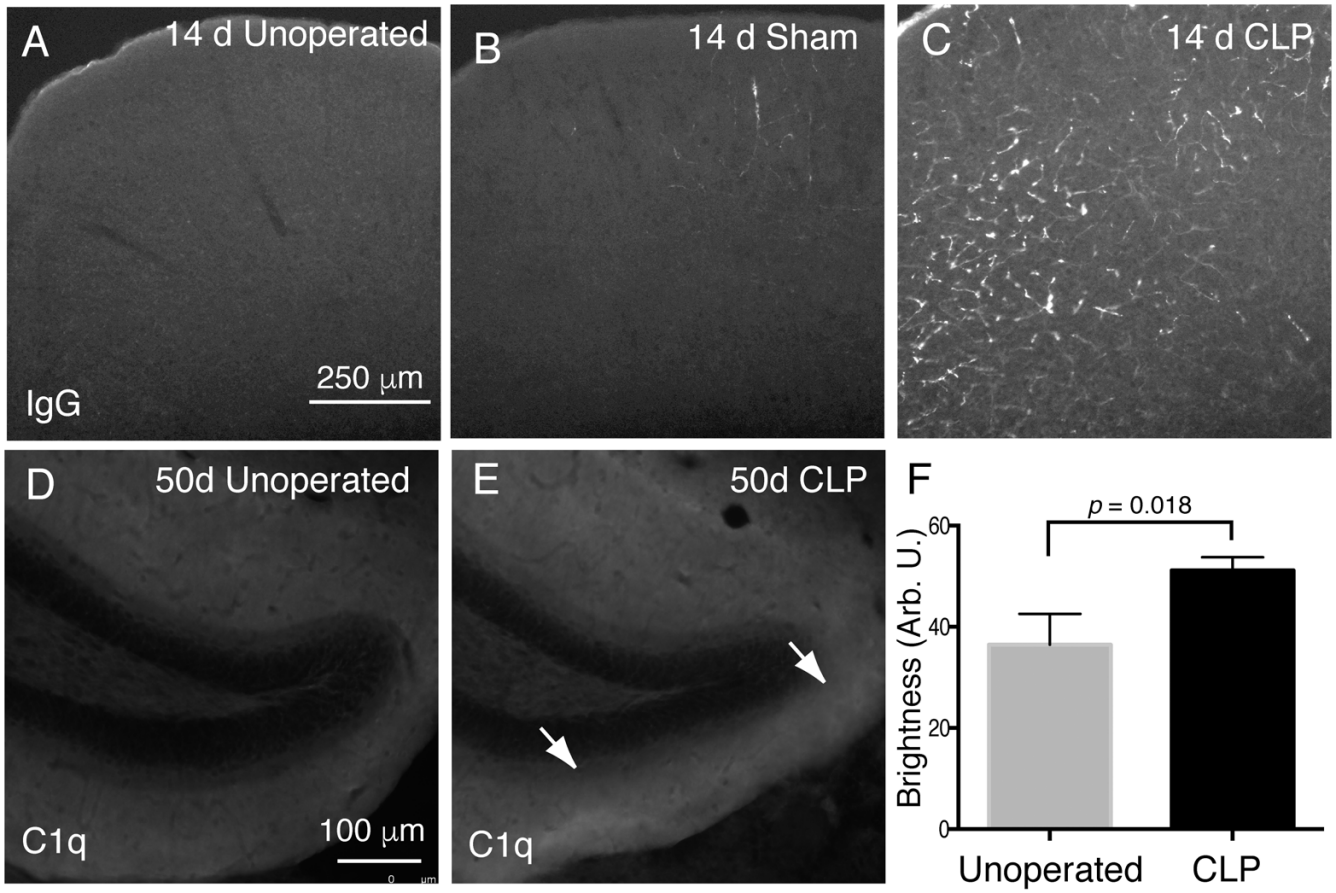
IgG and C1q deposition in the brain after CLP. Immunofluorescent staining for total mouse IgG does not reveal any tissue deposition in unoperated mice (A). In sham operated mice, there are sparse, focal areas of IgG immunoreactivity (B). After CLP, large areas of IgG immunoreactivity are present in a perivascular pattern (C). There is increased immunoreactivity for complement component C1q in the dentate gyrus in after CLP (arrows, E) compared to unoperated control mice (D, F).

While CLP does not result in significant structural changes in the hippocampus, we did note histologic evidence of an altered brain environment. CLP resulted in perivascular deposition of IgG, a marker of increased blood-brain barrier permeability [44] 14 days after sepsis. While there is no immunoreactivity for mouse IgG observed in unoperated mice (Fig 3A) and only scattered immunoreactivity visualized in sham operated controls (Fig 3B), post-CLP mice demonstrated abundant IgG immunoreactivity in a vascular pattern (Fig 3C). There was no immunoreactivity observed with anti-rabbit or anti-goat antibodies, indicating that the observed pattern was not due to nonspecific tissue damage (data not shown). Of note, this immunoreactivity was patchy, observed in both cortical and subcortical structures, without universal predilection for any particular structure, such as the hippocampus (not shown, n = 4 mice per group examined).

CLP was also associated with increased immunoreactivity for the complement component C1q, which may be blood borne and also produced within the CNS [37]. Immunoreactivity within the dentate gyrus was increased 50 days after CLP (Fig 3E) compared to age matched controls (Fig 3D and 3F). There was no immunoreactivity after staining with nonspecific rabbit IgG antibody (not shown). This increased immunoreactivity was confined to piriform cortex and the dentate gyrus, the regions of physiologic C1q expression, without notable ectopic or diffuse staining.

### CLP results in long-lasting myeloid infiltration of the CNS

**Fig 4:**
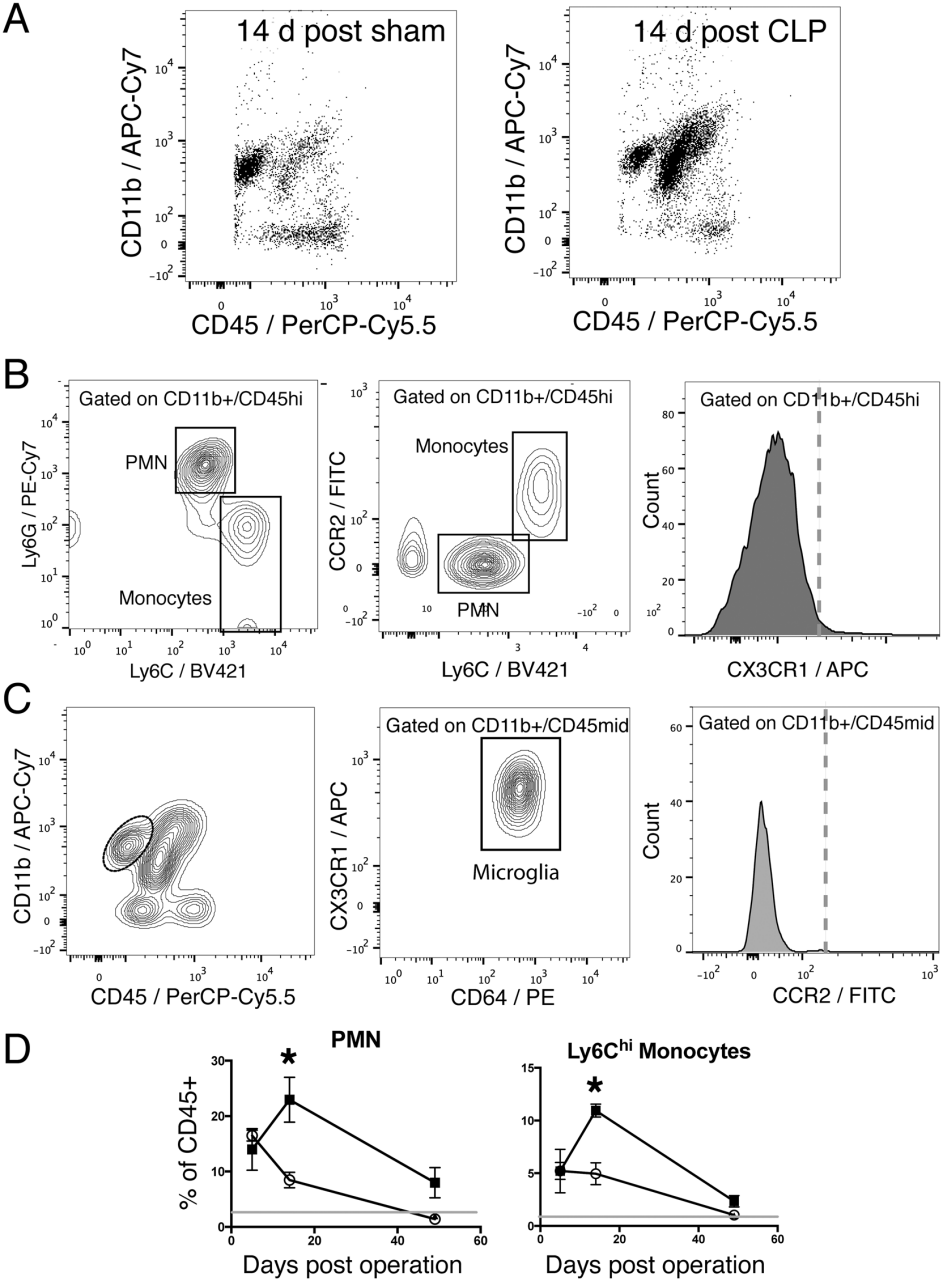
CLP results in long-lasting myeloid inflammation in the brain. A: 14 days after operation, an increased proportion of CD11b^+/^ CD45^hi^ cells are observed in the brains of post-CLP mice compared to sham controls. B: CD11b^+^/CD45^hi^ cells are divided into Ly6G^+^/Ly6C^mid^ and Ly6G^-^/Ly6C^hi^ subsets, corresponding to PMN and monocytes (left). Monocytes also express CCR2, while PMN do not (middle). Neither subset of CD11b^+^/CD45^hi^ cells express CX3CR1 (right, dashed line indicates isotype control intensity). C: CD11b^+^/CD45^mid^ cells (left, dotted circle) express the microglial markers CX3CR1 and CD64 (middle) and do not express CCR2 (right). D: Five days after CLP (filled squares) or sham operation (open circles), both groups demonstrate a small increase in PMN (left) and monocytes (right) compared to unoperated controls (gray line). The proportion of PMN and monocytes increase at 14 days in post-CLP mice, however, while decreasing in sham operated controls. By 50 days after CLP, the proportion of PMN and monocytes decreases nearly to baseline.

Given evidence of long-lasting behavioral changes and vasculopathy after sepsis, we studied the leukocyte composition of whole brain lysates 5 days, 14 days, and 50 days after sepsis. We found that post CLP animals had a dramatic increase in the proportion of CD45^hi^/CD11b^+^ myeloid cells 14 days after CLP when compared to sham operated controls (Fig 4A). This population of CD45^hi^/CD11b^+^ cells was principally composed of Ly6G^+^/Ly6C^mid^ neutrophils, and Ly6G^-^/Ly6C^hi^ monocytes (Fig 4B, left). Only Ly6C^hi^ cells expressed the monocyte marker CCR2 (Fig 4B, middle). CD45^hi^/CD11b^+^ cells were also negative for CX3CR1, and thus do not represent resident microglia which have upregulated CD45 expression in response to activation (Fig 4B, right). CD45^mid^/CD11b^+^ cells expressed the microglial markers CX3CR1 and CD64 [45] (Fig 4C) and were negative for CCR2 (Fig 4C). We did not observe any transitional cells expressing both CX3CR1 and CCR2.

The proportion of CD45^hi^ neutrophils and monocytes varied over time. At 5 days after operation, both post-CLP and post-sham animals had elevated numbers of neutrophils and monocytes compared to unoperated controls (Fig 4D). While the proportion of these cells declined over time in sham operated animals, the proportion of neutrophils and monocytes rose in post-CLP animals at 14 days, and was significantly different from sham controls at that time. By 50 days after CLP the proportion of neutrophils and monocytes in CLP and sham operated animals were not statistically significantly different when adjusted for multiple comparisons.

**Fig 5:**
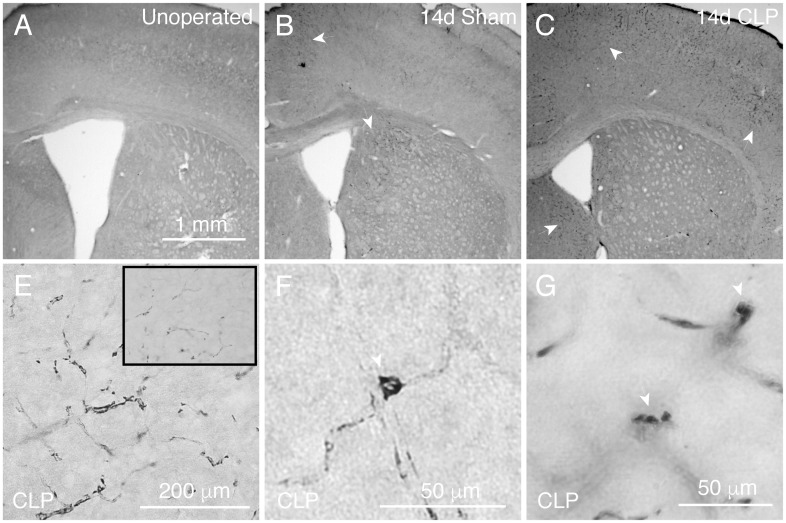
CLP results in patchy perivascular infiltration by monocytes. Unoperated mice do not demonstrate any CCR2 immunoreactivity in the brain (A). 14 days after sham operation, sham operated mice demonstrate sparse perivascular CCR2 immunoreactivity (B). CLP operated mice, however, demonstrate multiple cortical and subcortical dense patches of CCR2 immunoreactivity (C, white arrows). Highly immunoreactive cells include both elongated perivascular morphologies (E), extravasating cells (F), and punctate perivascular cells (G), all consistent with monocytes. Notable perivascular immunoreactivity is present in isotype stained tissue, likely reflecting cross-reactivity with nonspecific IgG deposition (E, inset).

It is important to note that the ratio of cell populations observed in brain lysates and studied by flow cytometry not only reflect the relative abundance of cell types *in situ*, but also the relative efficiency of cell isolation from tissue via homogenization and purification for each cell type. Therefore, it is unlikely that monocytes represent 10% of the total leukocyte population of the brain. We examined the distribution of monocytes *in situ* by immunohistochemistry for CCR2. We observed no immunoreactivity in unoperated controls (Fig 5A), and rare patchy areas of immunoreactivity in sham operated controls (Fig 5B). CLP operated controls demonstrated abundant CCR2 immunoreactivity in a patchy distribution in both cortical and subcortical structures (Fig 5C). Given that CLP also results in nonspecific IgG deposition in a similar distribution, we examined immunoreactivity to an isotype control antibody. Immunohistochemical staining with rat isotype control antibody suggested that much of the light staining in a perivascular pattern is due to nonspecific cross reactivity with mouse IgG (Fig 5E, inset). However, we did observe highly immunoreactive individual cells with elongated and rounded morphology in the perivascular space, representing monocytes (Fig 5D and 5G). CCR2+ cells were also observed extravasating from vessels (Fig 5F). Overall, immunohistochemical staining demonstrates the presence of monocytes in the perivascular spaces of the brain parenchyma in a scattered distribution in both cortical and subcortical areas that also display evidence of vasculopathy.

### CLP results in long-lasting elevation of CNS cytokines and chemokines

**Fig 6:**
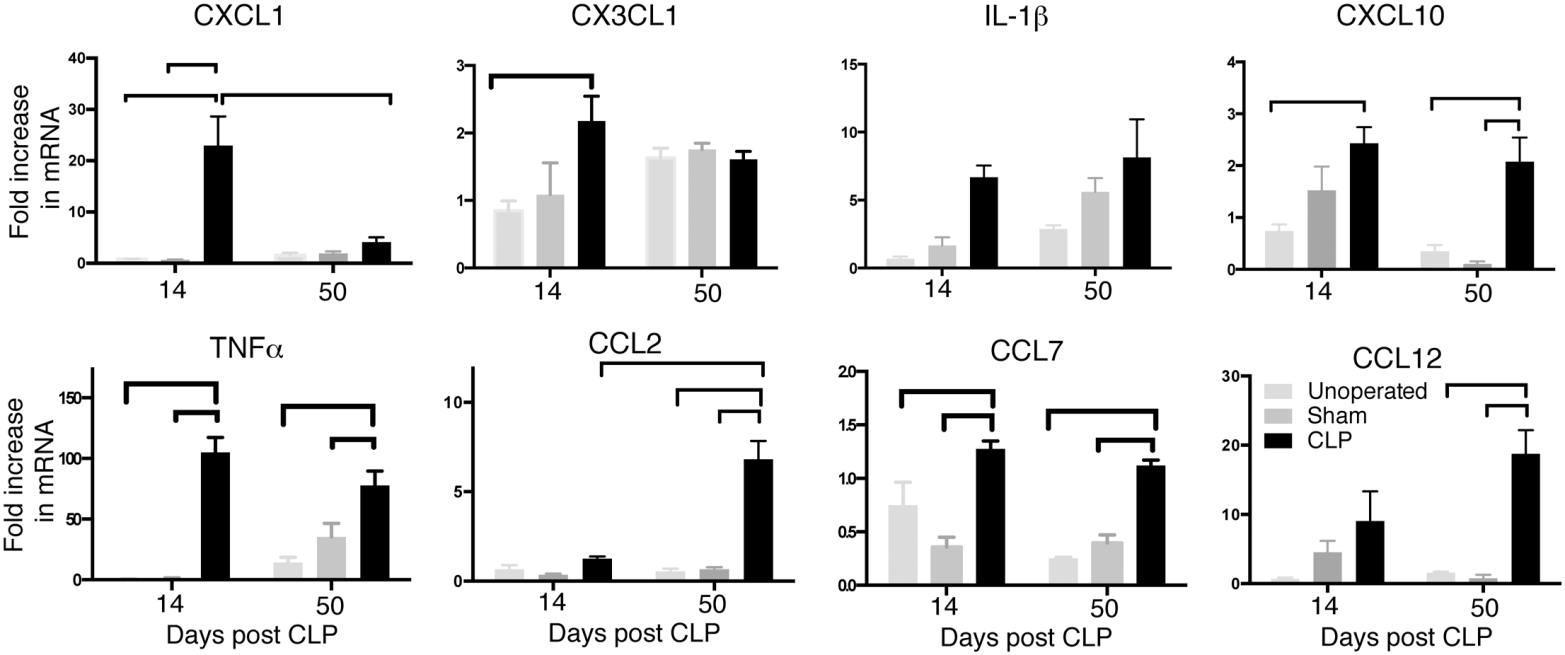
Gene expression measured from whole brain homogenates. Bars indicate differences with *p*<0.05 when corrected by the Bonferroni criterion for multiple comparisons.

Given that leukocyte infiltration to the brain continues to evolve for weeks following sepsis, we sought to understand whether CLP results in long-term expression of cytokines and chemokines within the CNS. RNA was isolated from whole brain homogenates 14 and 50 days after CLP, sham operation, and in age-matched controls. RT-PCR was performed for primary cytokines and chemokines (Fig 6). At 14 days after CLP, we observed significant increases in transcripts for the proximal cytokine TNFα, but not IL-1β. Transcripts of CX3CL1 (fractalkine), the ligand of the receptor CX3CR1 expressed on microglia, were moderately but significantly increased. Expression of the neutrophil chemokine CXCL1 and the monocyte chemokine CXCL10 were also increased two weeks after CLP, with a marked increase in CXCL1 expression. Expression of CXCL1 decreased by 50 days after CLP. We also examined transcripts for the monocyte/macrophage chemokines CCL2, CCL7, and CCL12, all ligands of the receptor CCR2. Surprisingly, expression of CCL2 and CCL12 increased from 14 to 50 days post CLP, rather than resolving (recheck stats for 14d expression of all 3). Transcripts of TNFα remained elevated 50 days post-CLP, as well.

### Recruitment of monocytes to the CNS is independent of CCR2

**Fig 7:**
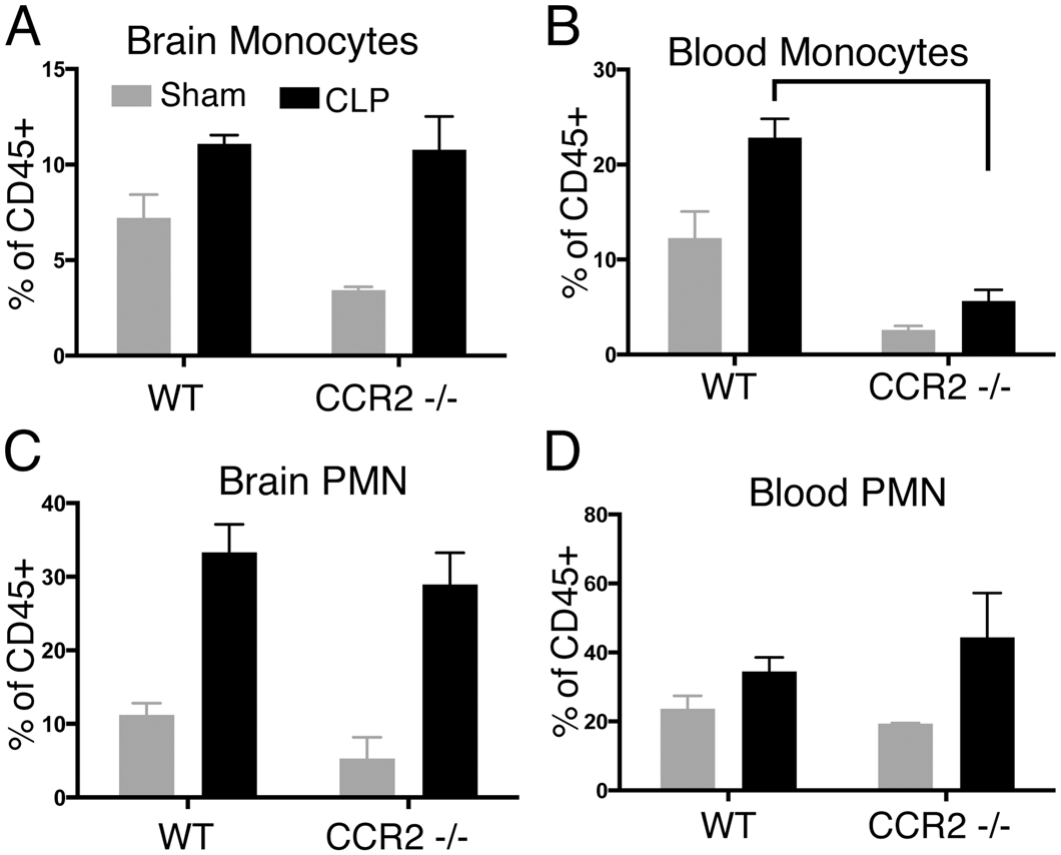
CNS myeloid inflammation is independent of peripheral monocytosis and CCR2 signaling. There was no change in the proportion of brain monocytes in CCR2^-/-^ mice after CLP (A). In contrast, CCR2 deficiency markedly decreases blood monocytosis (B). CCR2 deficiency has no effect on CNS (C) or blood (D) neutrophilia, as PMN do not express CCR2.

Given that transcription of the CCR2 ligands CCL2, CCL7, and CCL12 continued to increase even as the proportion of CCR2 expressing monocytes in the CNS decreased from 14 to 50 days, we sought to determine whether CCR2 mediated signaling was involved in recruitment of monocytes to the CNS, as it is in models of traumatic brain injury [46]. In addition, CLP not only results in infiltration of monocytes and neutrophils to the CNS, but systemic monocytosis and neutrophilia, as well, and CCR2 is important for exit of monocytes from the bone marrow (Fig 7B and 7D). Therefore, it is possible that myeloid infiltration into the CNS is a passive result of altered blood brain barrier permeability and high levels of circulating myeloid cells, rather than specific neuroinflammation. We tested these hypotheses by performing CLP and sham operation in *CCR2*^-/-^ mice [47]. Fourteen days after CLP, *CCR2*^-/-^ mice displayed a markedly decreased proportion of circulating monocytes compared to wild type mice, and no change in circulating neutrophils (Fig 7A and 7C). However, there was no significant change in the proportion of LyC^hi^ monocytes isolated from the CNS. This suggests that recruitment of monocytes to the CNS is a specific, tropic process. Moreover, despite the expression of CCR2 on Ly6C^hi^ monocytes and it importance for mobilization and trafficking in the periphery, recruitment of monocytes to the CNS may proceed in a CCR2 independent manner. CCR2 ligands, therefore, are likely subserving a process other than myeloid cell recruitment within the CNS.

### CLP results in changes in microglial gene expression in the absence of morphologic signs of activation

**Fig 8:**
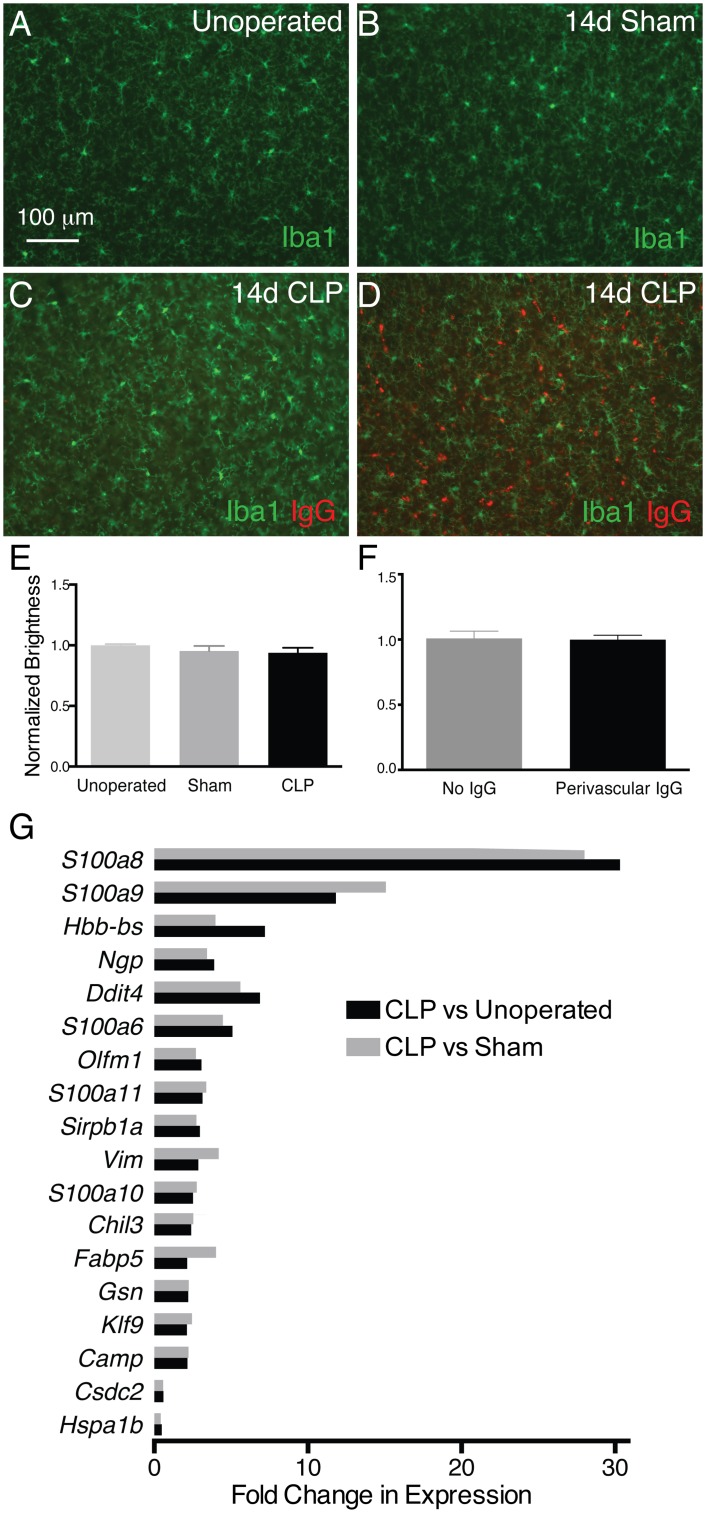
Microglia after CLP demonstrate distinct transcriptional differences, despite absence of morphological signs of activation. There is no difference in intensity of Iba1 immunoreactivity in somatosensory cortex among unoperated (A), sham operated (B) and post-CLP mice (C) 14 days after operation (E). There is also no difference in Iba1 immunoreactivity of microglia in regions of somatosensory cortex with evidence of vasculopathy (IgG deposition, D) compared to regions without IgG immunoreactivity (C, F). Despite the absence of activated amoeboid morphology, microglia in 14 day post-CLP mice demonstrate a distinct transcriptional profile compared to both sham operated and unoperated controls (G).

Our results indicate the presence of a myeloid cell infiltrate in the CNS for weeks after CLP, in addition to chronically elevated molecular markers of inflammation. Studies employing high dose LPS injection have demonstrated long-lasting morphologic changes in microglia, which are attributed to an activated pro-inflammatory state [48]. Immunofluorescence analysis for the microglial marker Iba1 14 days after CLP, however, did not demonstrate any significant morphologic changes in microglia of sepsis survivor animals or increased Iba1 immunoreactivity compared to sham operated or unoperated control mice (Fig 8A–8C and 8E). Immunofluorescence staining with nonspecific rabbit IgG antibody resulted in no immunoreactivity (not shown). Given we observed regional variations in IgG and CCR2 immunoreactivity, we examined the intensity of Iba1 immunoreactivity in regions without (Fig 8C) and with (Fig 8D) IgG deposition, and found no difference (Fig 8F).

Although amoeboid morphology is often taken as a surrogate for microglial activation [49], we hypothesized that given the evidence for a chronically altered brain environment in this model, CLP may result in changes to microglial phenotype without obvious morphologic changes [50]. We therefore isolated RNA from microglia (identified as CD11b^+^/CD45^mid^/CD64^+^ cells) in pooled brain homogenates (n = 3 animals per pool) of unoperated, sham, and CLP mice 14 days after operation. Gene expression was then examined via microarray analysis. Genes were considered differentially expressed if their expression differed at least 2-fold, and the robust multiarray average (RMA) expression value of at least one gene in the pair was 2^4^.

By this criterion, 97 genes were differentially regulated among CLP survivors and unoperated age-matched controls, and 86 genes were differentially regulated among CLP survivors and sham operated controls.

Fold change in gene expression for genes that were differentially expressed in CLP survivors compared to both sham and unoperated controls are shown (Fig 8G). These include multiple members of the S100A family, which have been implicated in chemotaxis, TLR4-mediated inflammation, as well as intracellular signaling in immune cells [51], as well as genes encoding antimicrobial proteins including neutrophilic granule protein and cathelicidin. Downregulated genes included cold and heat shock response genes. These data suggest that even in the absence of morphologic changes, sepsis has a long-lasting impact on microglial gene expression.

## Discussion

Cognitive decline and affective disorders are significant clinical problems contributing to functional disability in sepsis survivors. Numerous studies examine the acute neuroinflammatory response to sepsis, modeled either by CLP or high dose LPS in rodent models. Among studies which have examined the long-lasting CNS effects of sepsis survivorship in animal models, most have demonstrated long-lasting behavioral deficits and neuroinflammation using either high-dose LPS or CLP with high mortality, models which likely have limited fidelity to the physiology of patients with high rates of sepsis survival as indicated in epidemiologic studies.

Here, we employed CLP with antibiotic administration to achieve a model of sepsis with approximately 80% survival, similar to that observed in epidemiologic studies of sepsis survivors. While sepsis survivor animals did not demonstrate deficits in hippocampal dependent fear conditioning, survivors of this milder insult nonetheless had deficits in extinction learning. Extinction learning has been proposed as a rodent model of post traumatic stress disorder (PTSD), which has been reported in 25–35% of sepsis survivors [52,53]. Extinction learning also incorporates both cognitive and affective dimensions of behavior, and there is growing appreciation that sepsis survivor animals display increased anxiety and depressive-like behaviors [54,55]. Extinction learning and other affective behaviors are known to be altered by derangements in modulatory neurotransmission, and thus may be sensitive to a pro-inflammatory milieu that does not result in overt hippocampal cell death or alteration in dendritic structure [56–58]. While our analysis does not exclude structural changes in other brain regions or synaptic function, the behavioral and inflammatory changes observed occur in the absence of severe, diffuse cellular injury as might be expected from metabolic or hypoxic encephalopathy.

We found that sepsis survivor mice continue to experience significant neuroinflammation for weeks to months after CLP. This neuroinflammation does not appear to correlate to diffuse neuronal death or loss of synaptic density. Furthermore, we observed that brain levels of CCL2 and CCL12, ligands for CCR2, continue to increase even as the proportion of CNS CCR2^+^ cells diminished. This decoupling of chemokine expression and cellular infiltration suggests that these molecules may play a role beyond leukocyte chemoattraction to the brain. Indeed, we found that although CCR2 deficiency results in reduced trafficking of monocytes from the bone marrow to the periphery, as has been previously described, CCR2 expression was not necessary for CNS recruitment of Ly6C^hi^ monocytes. While CCL2 is elevated and monocytes are recruited to the brain acutely following systemic LPS injection and contribute to a pro-inflammatory environment in that model [12], other models have revealed varying relationships between CCR2 mediated signaling and cell recruitment in the CNS. In a model of traumatic brain injury, blockade of CCR2 signaling prevented recruitment of monocytes to the brain during a short window period following the injury [46]. In contrast, monocyte recruitment to the brain independent of CCR2 has been observed in *Listeria* encephalitis, a disease caused by an intracellular pathogen with tropism to the CNS [59]. Importantly, the behavioral phenotype reported here was observed 50 days after CLP, suggesting that brain dysfunction persists even as cellular infiltrates to the CNS are nearing resolution.

While cellular inflammation and infiltration likely do play important roles in brain injury and recovery after sepsis, the co-incidence of elevated cytokine and chemokine levels with a deficit in extinction learning raise the possibility that soluble inflammatory mediators affect neuronal function directly after sepsis. Certainly, cytokine signaling plays a role in a global inflammatory response and alters neurotrophic factors and other important signals in the brain environment [60]. However, chemokine and cytokine signaling may play a direct role in neurotransmission, especially by neuromodulatory neurotransmitters. CCL2 reduces the firing of serotonergic neurons when applied *ex vivo* [12]. Conversely, CCL2 increases levels of catecholamine synthetic enzymes [61] and increase release of dopamine [62] *in vivo*. Under conditions of dopamine excess after methamphetamine administration, TNFα decreases dopamine levels by increasing reuptake [63]. It is likely that complex networks of cytokines and chemokines acting directly on neurons during inflammatory states play a significant role in learning and behavior [64]. Studies examining the role of CCR2 ligands in this model are ongoing.

Studies examining the acute effects of LPS in the hours to days following administration suggest that microglia are dynamic producers of cytokines and regulators of the inflammatory environment [12,48]. Chronic neuroinflammation after a single dose of LPS also appears involve microglial and astrocytic activation [14,65]. Many studies of microglial activation use morphologic changes, with amoeboid morphology or increased immunoreactivity to Iba1, CD11b, or CD68 as markers of activation [49]. Moreover, these morphologic changes after systemic administration of LPS are often observed diffusely throughout the brain [66]. Here, we did not observe morphologic evidence of microglial activation. However, we did observe changes in gene expression at least 14 days following CLP. Though further work is required to establish the physiologic significance of these transcriptional changes, it is suggestive that the most highly upregulated genes in microglia after CLP are S100A8 and S100A9, which function as damage associated molecular patterns and may signal through both TLR4 and RAGE [67,68]. These DAMPs have also been implicated in acute cognitive changes and microgliosis in a model of postoperative neuroinflammation [69], and in neutrophil chemotaxis in bacterial meningitis [70]. Importantly, we did not observe upregulation of genes for TNFα, CXCL1, or CXCL10, or CCR2 ligand in the microglial gene expression analysis, suggesting that multiple cell types are responsible for the inflammatory signals detected in whole-brain homogenates.

The difference between these observations and the more typical amoeboid activation observed after LPS may be a result of altered kinetics of exposure to pro- and anti-inflammatory stimuli after CLP compared to a single high-dose administration of LPS. True infection exposes the immune system to varying levels of pathogen and damage associated molecular patterns (PAMPs and DAMPs) over hours to days. While more exposure to LPS may simply result in potentiation of the immune response [71], it is equally likely that varying levels of TLR4 agonism, even neglecting activation of other PAMP and DAMP receptors, result in alterations of downstream signaling that result in a balance between tolerance and priming of the innate immune response.

In addition to myeloid cell infiltration and microglial activation, we observed evidence of IgG deposition in a patchy vascular pattern 14 days after CLP. Both LPS and CLP may reduce the integrity of the blood-brain barrier and injure cerebrovascular endothelium in the acute setting [33]. While additional evidence is required to establish that this represents active extravasation of serum proteins through the blood brain barrier 2 weeks after sepsis and not just delayed clearance of protein leak from early on in the disease course [72], the presence IgG is suggestive of significant microvascular permeability and injury after CLP in this model, as well.

The chronicity of neuroinflammation, reflected in microvascular injury, myeloid cell infiltration, and cytokine expression, after sepsis suggests a reprogramming of the innate immune response. We speculate that a paracrine and autocrine signaling among multiple immunologically active cell types, including CNS resident microglia, bone marrow derived monocytes and neutrophils, and endothelium result in chronic neuroinflammation. This model is suggested by long-lasting elevation in cytokines even after the period of acute illness has passed. It is notable that both CCL2 and CXCL10, which are increased 50 days after CLP, have been shown *in vitro* to be induced by TNFα in immune and endothelial cells [73,74] and that TNFα also induces neuronal expression of these cytokines [75]. Conversely, CCR2 mediated signaling may potentiate release of TNFα from macrophages [76] and also contributes to dysfunction of the blood brain barrier [77]. TNFα signaling has also been shown to drive S100A8 and S100A9 expression during chronic inflammation [78]. Further work will focus on characterizing the cellular sources and interactions of the components of this putative cytokine network over time after CLP. Understanding the interaction between bone marrow derived inflammatory cells, the microvasculature, and resident microglia will provide and opportunity to disrupt this signaling network and potentially treat the reversible components of long-term cognitive decline after sepsis.
